# Antirheumatic drug response signatures in human chondrocytes: potential molecular targets to stimulate cartilage regeneration

**DOI:** 10.1186/ar2605

**Published:** 2009-02-03

**Authors:** Kristin Andreas, Thomas Häupl, Carsten Lübke, Jochen Ringe, Lars Morawietz, Anja Wachtel, Michael Sittinger, Christian Kaps

**Affiliations:** 1 Tissue Engineering Laboratory and Berlin – Brandenburg Center for Regenerative Therapies, Department of Rheumatology, Charité – Universitätsmedizin Berlin, Tucholskystrasse 2, 10117 Berlin, Germany; 2 Tissue Engineering Laboratory, Department of Rheumatology, Charité – Universitätsmedizin Berlin, Tucholskystrasse 2, 10117 Berlin, Germany; 3 University of Applied Sciences Wildau, Biosystems Technology, Bahnhofstrasse 1, 15745 Wildau, Germany; 4 Institute of Pathology, Charité – Universitätsmedizin Berlin, Charitéplatz 1, 10117 Berlin, Germany; 5 TransTissueTechnologies GmbH, Tucholskystrasse 2, 10117 Berlin, Germany

## Abstract

**Introduction:**

Rheumatoid arthritis (RA) leads to progressive destruction of articular cartilage. This study aimed to disclose major mechanisms of antirheumatic drug action on human chondrocytes and to reveal marker and pharmacological target genes that are involved in cartilage dysfunction and regeneration.

**Methods:**

An interactive *in vitro *cultivation system composed of human chondrocyte alginate cultures and conditioned supernatant of SV40 T-antigen immortalised human synovial fibroblasts was used. Chondrocyte alginate cultures were stimulated with supernatant of RA synovial fibroblasts, of healthy donor synovial fibroblasts, and of RA synovial fibroblasts that have been antirheumatically treated with disease-modifying antirheumatic drugs (DMARDs) (azathioprine, gold sodium thiomalate, chloroquine phosphate, and methotrexate), nonsteroidal anti-inflammatory drugs (NSAIDs) (piroxicam and diclofenac), or steroidal anti-inflammatory drugs (SAIDs) (methylprednisolone and prednisolone). Chondrocyte gene expression profile was analysed using microarrays. Real-time reverse transcription-polymerase chain reaction and enzyme-linked immunosorbent assay were performed for validation of microarray data.

**Results:**

Genome-wide expression analysis revealed 110 RA-related genes in human chondrocytes: expression of catabolic mediators (inflammation, cytokines/chemokines, and matrix degradation) was induced, and expression of anabolic mediators (matrix synthesis and proliferation/differentiation) was repressed. Potential marker genes to define and influence cartilage/chondrocyte integrity and regeneration were determined and include already established genes (*COX-2*, *CXCR-4*, *IL-1RN*, *IL-6/8*, *MMP-10/12*, and *TLR-2*) and novel genes (*ADORA2A*, *BCL2-A1*, *CTGF*, *CXCR-7*, *CYR-61*, *HSD11B-1*, *IL-23A*, *MARCKS*, *MXRA-5*, *NDUFA4L2*, *NR4A3*, *SMS*, *STS*, *TNFAIP-2*, and *TXNIP*). Antirheumatic treatment with SAIDs showed complete and strong reversion of RA-related gene expression in human chondrocytes, whereas treatment with NSAIDs and the DMARD chloroquine phosphate had only moderate to minor effects. Treatment with the DMARDs azathioprine, gold sodium thiomalate, and methotrexate efficiently reverted chondrocyte RA-related gene expression toward the 'healthy' level. Pathways of cytokine-cytokine receptor interaction, transforming growth factor-beta/Toll-like receptor/Jak-STAT (signal transducer and activator of transcription) signalling and extracellular matrix receptor interaction were targeted by antirheumatics.

**Conclusions:**

Our findings indicate that RA-relevant stimuli result in the molecular activation of catabolic and inflammatory processes in human chondrocytes that are reverted by antirheumatic treatment. Candidate genes that evolved in this study for new therapeutic approaches include suppression of specific immune responses (*COX-2*, *IL-23A*, and *IL-6*) and activation of cartilage regeneration (*CTGF *and *CYR-61*).

## Introduction

Progressive destruction of articular structures and chronic inflammation of synovial joints are major pathophysiological outcomes of rheumatoid arthritis (RA) [1]. As the disease progresses, destruction of joint cartilage and, eventually, loss of joint function cause excessive morbidity and disability. Current approaches to drug therapy for RA focus predominantly on the alleviation of inflammation, pain, and disease progression. Among the medicinal strategies, nonbiological disease-modifying antirheumatic drugs (DMARDs) (for example, azathioprine, gold sodium thiomalate, chloroquine phosphate, and methotrexate [MTX]), steroidal anti-inflammatory drugs (SAIDs) (for example, prednisolone and methylprednisolone), and nonsteroidal anti-inflammatory drugs (NSAIDs) (for example, piroxicam and diclofenac) have already been successfully employed. The new group of biologics specifically targets inflammatory cytokines (for example, tumour necrosis factor [TNF] inhibitor etanercept) or receptors [2,3].

Despite recent progress in controlling inflammation, little cartilage repair has yet to be observed. Probably, suppression of inflammation is not sufficient to restore joint structure and function, and significant cartilage repair may be achieved only by activation of local chondrocyte regeneration [4]. This underlines the need to identify distinct genes of RA-related chondrocyte dysfunction and to elucidate potential molecular mechanisms, markers, and pharmacological targets in human chondrocytes that might be involved in cartilage regeneration and suppression of inflammation. Gene expression profiling may be of help here to offer a better molecular understanding of chondrocyte dysfunction and regeneration and to disclose new therapeutic strategies [5].

Key mediators of joint destruction are RA synovial fibroblasts (RASFs), which directly destroy cartilage by secreting matrix-degrading enzymes [6,7]. Numerous studies on the gene expression and protein secretion of RASFs have elucidated potent diagnostic and therapeutic targets in RASFs that mediate direct joint destruction and inflammation [8-13]. Recent studies have offered insight into the mechanisms of drug action; the molecular effects on RASFs following treatment with frequently used antirheumatic drugs were determined by genome-wide expression profiling [14].

Beyond direct cartilage destruction, RASFs maintain inflammation in synovial joints and induce chondrocyte dysfunction by releasing proinflammatory cytokines, in particular TNF-alpha and interleukin (IL)-1-beta, and catabolic mediators [6,15]. Inflammatory and catabolic stimuli from RASFs cause indirect cartilage destruction; a disturbed tissue homeostasis and a shift to catabolic mechanisms lead to suppressed matrix synthesis and induce the production of degradative mediators by chondrocytes, such as matrix metalloproteinases (MMPs), prostaglandins, and nitric oxide [16,17]. Recently, we determined the RASF-induced expression profile in human chondrocytes that disclosed genes that are related to cartilage destruction and that involve marker genes of inflammation/nuclear factor-kappa-B (NF-κB) signalling, cytokines, chemokines and receptors, matrix degradation, and suppressed matrix synthesis [18]. Although much is known about RASFs as key mediators of cartilage destruction in RA, researchers have scarcely analysed the molecular mechanisms of cartilage regeneration induced by antirheumatic treatment. Thus, the aim of this study was to establish an interactive *in vitro *model that comprehensively illustrates the diversity of antirheumatic drug effects on human chondrocytes and that offers the opportunity for parallel and future drug testing. To reveal marker and target genes for stimulation of cartilage/chondrocyte regeneration and suppression of inflammation was an additional goal of this study.

In the present study, human chondrocytes were cultured in alginate beads and were stimulated with supernatant of RASFs, healthy donor synovial fibroblasts (NDSFs), and drug-treated RASFs, respectively. Genome-wide microarray analysis was performed to determine RA-related gene expression and antirheumatic drug response signatures in human chondrocytes. Real-time reverse transcription-polymerase chain reaction (RT-PCR) and enzyme-linked immunosorbent assay (ELISA) were performed for validation of microarray data.

## Materials and methods

### Cell culture

The local ethics committee of the Charité Berlin approved this study.

#### Human chondrocytes

Healthy human articular cartilage was obtained from knee condyles of donors *post mortem *(n = 6 donors, age range of 39 to 74 years and mean age of 60 years) without known predisposing conditions for joint disorders. No macroscopic signs of cartilage degradation or traumatic alterations were present. Human chondrocytes were harvested as described previously [19] and expanded in monolayer culture. Reaching confluence, chondrocytes were detached with 0.05% trypsin/0.02% ethylenediaminetetraacetic acid (EDTA) (Biochrom AG, Berlin, Germany) and subcultured at 10,000 cells per centimetre squared. Reaching confluence again, human chondrocytes were trypsinised, encapsulated in alginate beads at 2 × 10^7 ^cells per millilitre in 1.5% (wt/vol) alginate (Sigma-Aldrich, Munich, Germany) as described previously [18], and three-dimensionally cultured for 14 days.

#### Synovial fibroblasts

Human SV40 T-antigen immortalised synovial fibroblasts (SFs) were derived from primary synovial cells that were obtained from synovial pannus tissue of an RA patient by surgical synovectomy (RASFs, HSE cell line) and from normal (healthy) donor synovial tissue following meniscectomy (NDSFs, K4IM cell line). RASFs represent a prototype of activated SFs [20,21], and NDSFs represent healthy SFs [22].

Chondrocyte alginate beads and SFs were cultured separately in RPMI 1640 (Biochrom AG) supplemented with 10% human serum (German Red Cross, Berlin, Germany), 100 ng/mL amphotericin B, 100 U/mL penicillin, 100 μg/mL streptomycin (Biochrom AG), and 170 μM ascorbic acid 2 phosphate (Sigma-Aldrich).

### MTS cytotoxicity assay

Cytotoxic effects of antirheumatic drugs on RASFs were determined by MTS (3-[4,5-dimethylthiazol-2-yl]-5-[3-carboxymethoxyphenyl]-2-[4-sulfophenyl]-2H-tetrazolium) cell proliferation assay (Promega GmbH, Mannheim, Germany). SFs were seeded at a density of 3 × 10^3 ^cells per well into 96-well plates in triplicate. Reaching 70% confluence, medium was replaced by phenol red-free RPMI 1640 medium (Biochrom AG) containing azathioprine (0 to 400 μg/mL, Imurek; GlaxoSmithKline GmbH, Munich, Germany), gold sodium thiomalate (0 to 100 μg/mL, Tauredon; Altana Pharma Deutschland GmbH, Konstanz, Austria), chloroquine phosphate (0 to 400 μg/mL, Resochin; Bayer Vital GmbH, Leverkusen, Germany), MTX (0 to 10 μg/mL, Methotrexat; Medac GmbH, Hamburg, Germany), piroxicam (0 to 400 μg/mL, pirox-ct; CT-Arzneimittel GmbH, Berlin, Germany), diclofenac (0 to 200 μg/mL, Diclofenac; ratiopharm GmbH, Ulm, Germany), methylprednisolone (0 to 2,000 μg/mL, Urbason; Aventis Pharma Deutschland GmbH, Frankfurt am Main, Germany), or prednisolone (0 to 2,000 μg/mL, Solu Decortin H; Merck, Darmstadt, Germany). Control cultures were maintained in phenol red-free medium without drug supplementation. Following 48 hours of drug treatment, MTS assay was performed according to the instructions of the manufacturer. Drug concentrations that resulted in 80% metabolic activity of RASFs compared with untreated controls (20% inhibitory concentration [IC_20_]) were determined. Drug-treated synovial cells were assessed microscopically for typical fibroblast-like morphology.

### Experimental setup

**Figure 1:**
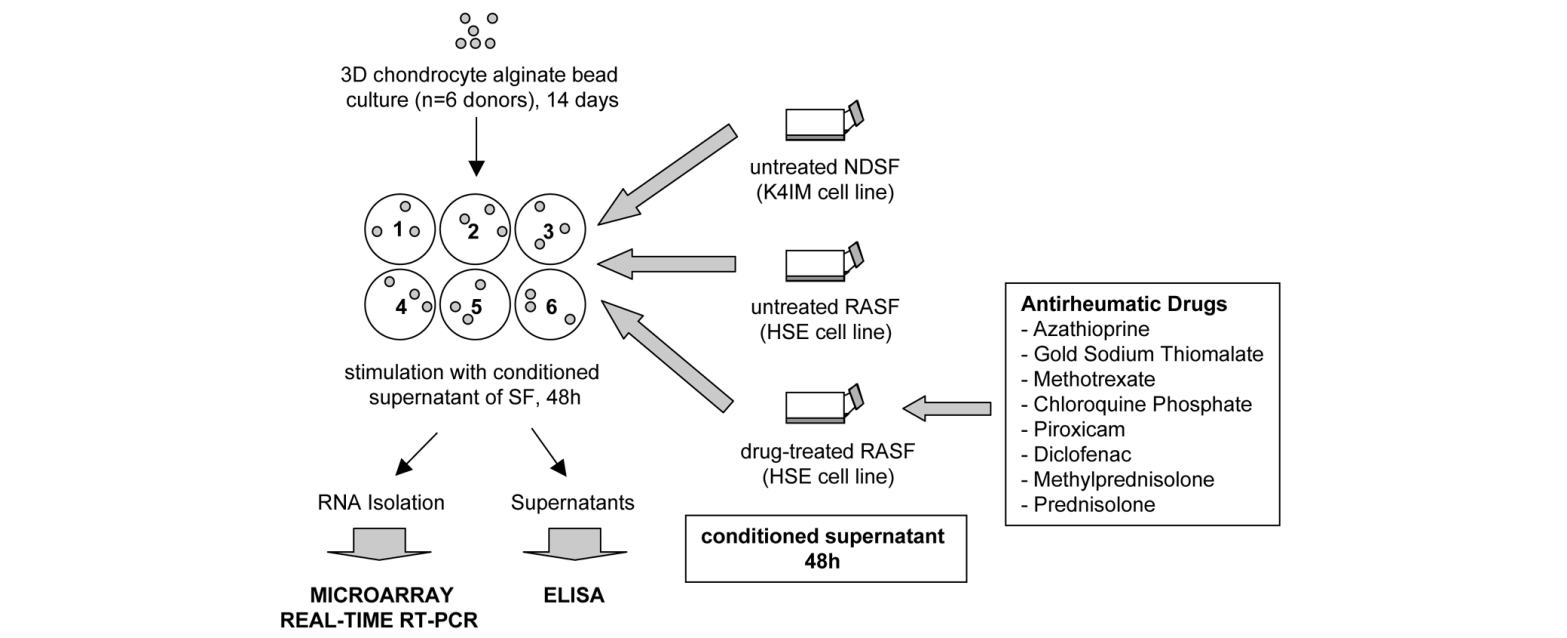
Experimental setup. Medium of subconfluent normal (healthy) donor synovial fibroblasts (NDSFs) and rheumatoid arthritis synovial fibroblasts (RASFs) was conditioned for 48 hours. RASFs were incubated for 48 hours with medium containing a 20% inhibitory concentration of antirheumatic drugs. Cartilage-like alginate beads (n = 6 donors) were stimulated for 48 hours with conditioned supernatant of untreated RASFs, untreated NDSFs, and drug-treated RASFs, respectively. Following interactive cultivation, isolation of total RNA was performed and chondrocyte supernatants were collected. Genome-wide expression profiling, real-time reverse transcription-polymerase chain reaction (RT-PCR), and enzyme-linked immunosorbent assay (ELISA) analysis were performed. 3D, three-dimensional; SF, synovial fibroblast.

Figure 1 illustrates the setup of the conducted experiments. Medium of subconfluent NDSFs and RASFs was conditioned for 48 hours. RASFs were incubated for 48 hours with medium containing IC_20 _of azathioprine, gold sodium thiomalate, chloroquine phosphate, MTX, piroxicam, diclofenac, methylprednisolone, and prednisolone, respectively. Cartilage-like alginate beads (n = 6 donors) were stimulated for 48 hours with conditioned supernatant of untreated NDSFs (NDSFsn), of untreated RASFs (RASFsn), and of drug-treated RASFs. Following interactive cultivation, isolation of total RNA was performed and supernatants were collected. Genome-wide expression profiling, real-time RT-PCR, and ELISA were conducted.

### RNA isolation and genome-wide expression profiling

Stimulated human chondrocytes were harvested from alginate beads as described previously [18]. In brief, alginate beads were solubilised on ice and human chondrocytes were harvested by centrifugation. Total RNA was isolated using an RNeasy Mini Kit (Qiagen, Hilden, Germany) in accordance with the instructions of the manufacturer. In addition, proteinase K and DNase I digestions were performed. Isolation of total RNA was performed for each donor separately (n = 6 donors). Equal amounts of total RNA from three different donors were pooled, yielding two different experimental groups (two pools with three donors for each pool) for untreated controls and for each drug treatment. Pooled RNA was used for microarray analysis and for real-time RT-PCR. Microarray analysis was performed using the oligonucleotide microarray HG U133A GeneChip (Affymetrix, High Wycombe, UK) in accordance with the recommendations of the manufacturer. In brief, 2.5 μg of pooled RNA was used to generate biotin-labelled cRNA by cDNA synthesis and *in vitro *transcription. Next, 10 μg (50 μg/mL) of fragmented cRNA was hybridised to the oligonucleotide microarrays, and GeneChips were washed, stained, and scanned as recommended.

### Microarray data mining

Raw gene expression data analyses were processed using (a) GeneChip Operating Software (GCOS) (Affymetrix) and (b) Robust Multichip Analysis (RMA) [23]. Genes were differentially expressed if regulated greater than or equal to twofold or less than or equal to twofold as determined by both GCOS and RMA statistical analyses in both experimental groups (two pools with three donors for each pool). Microarray data mining was performed in accordance with the procedure described in Table 1. First, RA-related genes and pathways were identified in human chondrocytes. For this purpose, differentially expressed genes were determined between RASFsn-stimulated chondrocytes ('diseased' status) and NDSFsn-stimulated chondrocytes ('healthy' status). These genes were considered to be relevant to chondrocyte dysfunction in RA. Next, expression levels of the determined RA-related genes were analysed following treatment with antiheumatic drugs. The antirheumatic drug response signatures were supposed to comprise all RA-related genes that were reverted by treatment from the 'diseased' expression level in RASFsn-stimulated chondrocytes toward the 'healthy' level in NDSFsn-stimulated chondrocytes. These marker genes were considered to be relevant for drug-induced cartilage/chondrocyte regeneration and suppression of inflammation.

**Table 1:** Microarray data mining

Analysis	Finding
RA-related genes in human chondrocytes differentially expressed in human chondrocytes that were stimulated with supernatant of RASFs versus NDSF stimulation	- 110 pharmacological marker genes and relevant pathways of RA-related chondrocyte dysfunction
	
KEGG pathway analysis	
	
Antirheumatic drug response signatures in human chondrocytes	- Mechanism of drug action
Differential expression of RA-related genes in human chondrocytes due to antirheumatic treatment of RASFs (stimulation of human chondroctyes with supernatant of drug-treated RASFs versus stimulation with supernatant of untreated RASFs)	- 94 pharmacological marker genes and relevant pathways for stimulation of cartilage regeneration and suppression of inflammation
	
Hierarchical clustering analysis, principal components analysis, and KEGG pathway analysis	
	
Validation of microarray data	- Microarray data were confirmed for selected genes/proteins
- Real-time reverse transcription-polymerase chain reaction	
- Enzyme-linked immunosorbent assay	

KEGG, Kyoto Encyclopaedia of Genes and Genomes; NDSF, healthy donor synovial fibroblast; RA, rheumatoid arthritis; RASF, rheumatoid arthritis synovial fibroblast.

To visualise and to compare the RA-related chondrocyte gene expression pattern for the different therapies, hierarchical cluster and principal components analyses with normalised mean gene expression values were performed with Genesis 1.7.2 software (Graz University of Technology, Institute for Genomics and Bioinformatics, Graz, Austria) [24]. Functional annotation was determined according to reports from the literature. Pathway analysis was performed to disclose relevant mechanisms that are related to chondrocyte dysfunction in RA and to drug-induced chondrocyte regeneration and suppression of inflammation. For this purpose, expression levels of RA-related genes were submitted to the Database for Annotation, Visualisation, and Integrated Discovery (DAVID) and to the Kyoto Encyclopaedia of Genes and Genomes (KEGG) database [25,26]. Determined KEGG pathways showed a *P *value of less than or equal to 0.05. Microarray data have been deposited in the National Center for Biotechnology Information Gene Expression Omnibus (GEO) and are accessible through GEO series accession number [GEO:GSE12860].

### Real-time reverse transcription-polymerase chain reaction

Expression of selected genes was verified by real-time RT-PCR. Pooled total RNA (two pools with three donors for each pool) was reverse-transcribed with an iScript cDNA synthesis kit as recommended by the manufacturer (Bio-Rad Laboratories GmbH, Munich, Germany). TaqMan real-time RT-PCR was performed in triplicates in 96-well optical plates on an ABI Prism 7700 Sequence Detection System (Applied Biosystems, Darmstadt, Germany) using primer and probe sets from Applied Biosystems for cyclooxygenase 2 (*COX-2*, Hs00153133_m1), chemokine (C-X-C motif) receptor 4 (*CXCR-4*, assay ID Hs00607978_s1), thioredoxin interacting protein (*TXNIP*, Hs00197750_m1), steroid sulfatase (*STS*, Hs00165853_m1), and glyceraldehyde 3-phosphate dehydrogenase (*GAPDH*, Hs99999905_m1). The endogenous expression level of *GAPDH *was used to normalise gene expression levels, and relative quantification of gene expression was given as a percentage of *GAPDH*.

### Enzyme-linked immunosorbent assay

Supernatants were collected and stored at -20°C. Levels of IL-6, CXCL-8 (IL-8), and CCL-20 (macrophage inflammatory protein-3-alpha, or MIP-3α) were measured using quantitative sandwich enzyme immunoassay (ELISA) in accordance with the recommended procedures of the manufacturer (RayBiotech, Inc., Norcross, GA, USA). Background signals of SF supernatants were subtracted, and protein concentration was normalised to one chondrocyte alginate bead. For statistical analysis, *t *test (normal distribution) or Mann-Whitney rank sum test (non-normal distribution) was applied using Sigmastat software (Systat Software, San Jose, CA, USA).

## Results

### Cytotoxicity of antirheumatic drugs on rheumatoid arthritis synovial fibroblasts

For standardisation of this study and to ensure cell viability and drug response, the effective doses of the examined antirheumatic drugs on RASFs were determined. By means of cytotoxicity assays, drug concentrations that resulted in 80% vitality of RASFs following 48 hours of drug exposure compared with untreated controls were identified. The following IC_20 _values were determined: 10 μg/mL azathioprine, 5 μg/mL gold sodium thiomalate, 50 μg/mL chloroquine phosphate, 0.2 μg/mL MTX, 25 μg/mL piroxicam, 75 μg/mL diclofenac, 1 μg/mL methylprednisolone, and 1 μg/mL prednisolone (data not shown). The typical fibroblast-like morphology of RASFs was maintained following treatment with these drug concentrations (data not shown). The respective IC_20 _drug concentrations were applied for antirheumatic treatment of RASFs in the subsequent experiments.

### Rheumatoid arthritis-related gene expression in human chondrocytes

For identification of RA-related changes, differentially expressed genes were determined in human chondrocytes that have been stimulated with supernatant of RASFs ('diseased' status) compared with NDSF stimulation ('healthy' status). This revealed 110 genes that are involved in inflammation/NF-κB signalling pathway, cytokines/chemokines and receptor interaction, immune response, proliferation/differentiation, matrix degradation, and suppressed matrix synthesis (Additional data files 1 and 2). Genes that are known to be associated with immunological processes (inflammation [for example, *ADORA2A*, *IL-1RN*, *TLR-2*, and *COX-2*] and cytokines/chemokines [for example, *IL-23A*, *CXCR-4/7*, *CCL-20*, and *CXCL-1–3/8*]) or catabolic mechanisms (matrix degradation [for example, *MMP-10/12*]) were induced, and anabolic mediators (matrix synthesis [for example, *VCAN*] and proliferation/differentiation [for example, *WISP-2 *and *CTGF*]) were repressed. Thus, these 110 genes demonstrated a disturbed chondrocyte homeostasis and respective genes were considered to be relevant for chondrocyte dysfunction in RA.

### Antirheumatic drug response signatures in human chondrocytes and genes to define and influence cartilage integrity and regeneration

**Figure 2:**
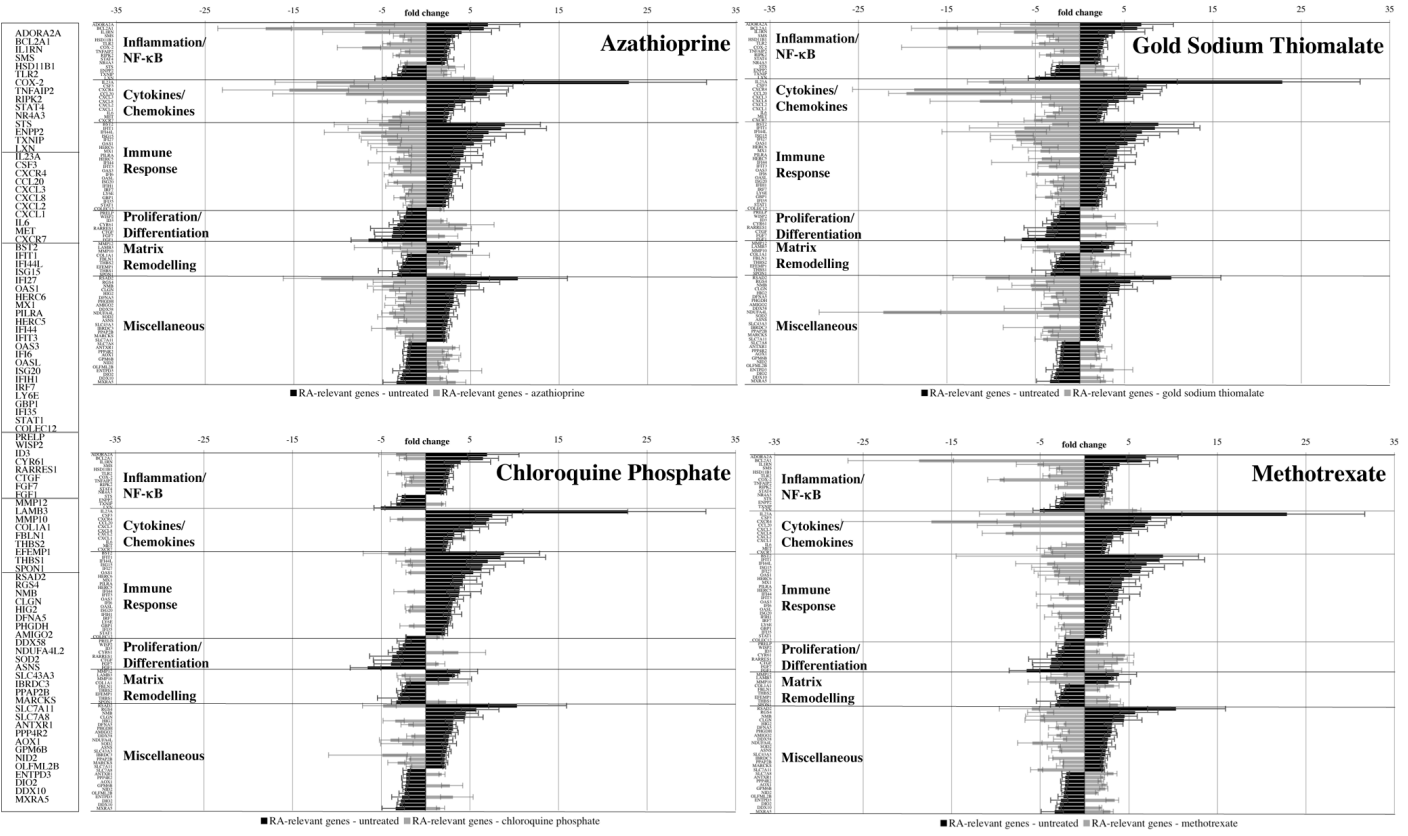
Disease-modifying antirheumatic drug (DMARD) response signatures in human chondrocytes. Centroid view (fold change) of rheumatoid arthritis (RA)-related chondrocyte gene expression following treatment of rheumatoid arthritis synovial fibroblasts (RASFs) with DMARDs azathioprine, gold sodium thiomalate, chloroquine phosphate, and methotrexate. Black bars represent the RA-related gene expression in human chondrocytes (differential gene expression of RASFsn-stimulated chondrocytes versus NDSFsn stimulation). Grey bars represent the DMARD response signatures in human chondrocytes (differential gene expression of human chondrocytes stimulated with drug-treated RASFs compared with stimulation with untreated RASFs). Azathioprine, gold sodium thiomalate, and methotrexate treatment of RASFs resulted in a reverted gene expression of the majority of RA-related genes in human chondrocytes. In contrast, RASF treatment with chloroquine phosphate had only minor effects. NDSFsn, supernatant of untreated normal (healthy) donor synovial fibroblast; NF-κB, nuclear factor-kappa-B; RASFsn, supernatant of untreated rheumatoid arthritis synovial fibroblast.

**Figure 3:**
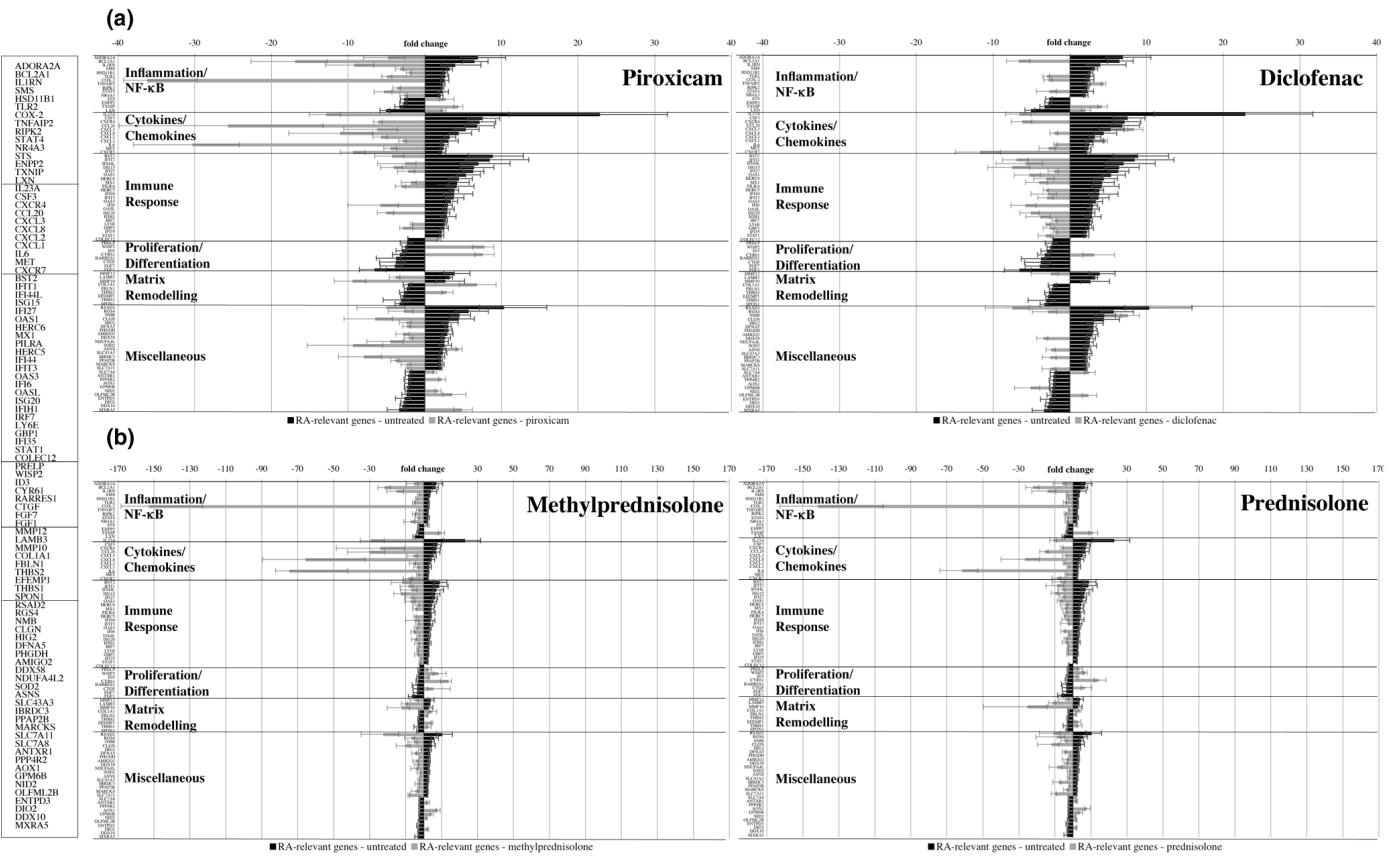
Nonsteroidal anti-inflammatory drug (NSAID) and steroidal anti-inflammatory drug (SAID) response signatures in human chondrocytes. Centroid view (fold change) of rheumatoid arthritis (RA)-related chondrocyte gene expression following treatment of rheumatoid arthritis synovial fibroblasts (RASFs) with **(a) **NSAIDs piroxicam and diclofenac and **(b) **SAIDs methylprednisolone and prednisolone. Black bars represent the RA-related gene expression in human chondrocytes (differential gene expression of RASFsn-stimulated chondrocytes versus NDSFsn stimulation). Grey bars represent the NSAID/SAID response signatures in human chondrocytes (differential gene expression of human chondrocytes stimulated with drug-treated RASFs compared with stimulation with untreated RASFs). Whereas piroxicam mainly influenced the expression of RA-related genes involved in inflammation/nuclear factor-kappa-B (NF-κB) and cytokines/chemokines, diclofenac predominantly had an impact on the expression of genes associated with immune response. Expression of numerous RA-related genes was not influenced by NSAID treatment. In contrast, SAID treatment led to an almost complete reversion of chondrocyte RA-related gene expression. The expression of distinct genes involved in inflammation and cytokines/chemokines (*BCL2-A1*, *COX-2*, *CXCL-8/IL-8*, and *IL-6*) was strongly repressed. NDSFsn, supernatant of untreated healthy donor synovial fibroblast; RASFsn, supernatant of untreated rheumatoid arthritis synovial fibroblast.

For identification of major mechanisms and molecular markers and targets of chondrocyte regeneration, RASFs were treated with different antirheumatic drugs and conditioned supernatants were used for chondrocyte stimulation. The antirheumatic drug response signatures were investigated for the determined 110 RA-related genes in human chondrocytes to characterise the drug-related reversion from the 'diseased' expression level toward the 'healthy' level. Expression of 94 genes was reverted by at least one type of treatment (Additional data file 1, Figures 2 and 3). Response to treatment suggests that these genes also reflect molecular processes relevant for therapeutic interference to maintain and regenerate cartilage. Apart from known marker genes of cartilage/chondrocyte integrity and regeneration (*COX-2*, *CXCR-4*, *IL-1RN*, *IL-6/8*, *MMP-10/12*, and *TLR-2*), numerous novel markers, including *ADORA2A*, *BCL2-A1*, *CTGF*, *CXCR-7*, *CYR-61*, *HSD11B-1*, *IL-23A*, *MARCKS*, *MXRA-5*, *NDUFA4L2*, *NR4A3*, *SMS*, *STS*, *TNFAIP-2*, and *TXNIP*, were determined. On the contrary, the expression of the 16 remaining RA-related chondrocyte genes was not reverted by treatment with any of the antirheumatic drugs examined (Additional data file 2). These genes include phospholipase A2 group IIA (*PLA2G2A*), chondroitin sulfate proteoglycan 2 (*VCAN*), and pentraxin-related gene (*PTX3*).

#### Treatment with disease-modifying antirheumatic drugs

When exposing RASFs to DMARDs (Figure 2), azathioprine, gold sodium thiomalate, and MTX efficiently reverted the RA-induced molecular changes in chondrocytes toward the 'healthy' level; in particular, genes related to inflammation/NF-κB pathway, cytokine/chemokine activity, immune response, proliferation/differentiation, and matrix remodelling were involved. In contrast, only a minority of RA-related changes were reverted by treatment with chloroquine phosphate. Thus, to reconstitute the molecular signature of cartilage/chondrocytes, azathioprine, gold sodium thiomalate, or MTX seem to be much more effective than chloroquine phosphate.

#### Treatment with nonsteroidal anti-inflammatory drugs

Treatment of RASFs with NSAIDs (piroxicam and diclofenac) reverted the expression of approximately 50% of the RA-induced changes in human chondrocytes (Figure 3a). Exposure of RASFs to piroxicam predominantly regulated expression of genes in chondrocytes that are related to inflammation/NF-κB pathway and cytokines/chemokines. In contrast, diclofenac treatment reverted expression of genes predominantly associated with immune response. However, numerous other RA-induced changes were not affected by NSAID treatment, and thus treatment of RASFs with NSAIDs showed only moderate effects on chondrocytes.

#### Treatment with steroidal anti-inflammatory drugs

After treatment of RASFs with SAIDs (methylprednisolone and prednisolone), a nearly complete and very efficient reversion from the 'diseased' toward the 'healthy' level was determined in human chondrocytes (Figure 3b). Thus, genes of all six functional annotation groups were involved and several genes (*Bcl2-related protein A1 *[*BCL2-A1*], *COX-2*, *chemokine (C-X-C motif) ligand-8 *[*CXCL-8/IL-8*], and *IL-6*) were reverted even beyond the level of controls stimulated with NDSF supernatant. In addition, methylprednisolone and prednisolone treatment of RASFs showed very similar effects on the RA-related gene expression pattern in human chondrocytes.

### Quantification of drug effects

**Figure 4:**
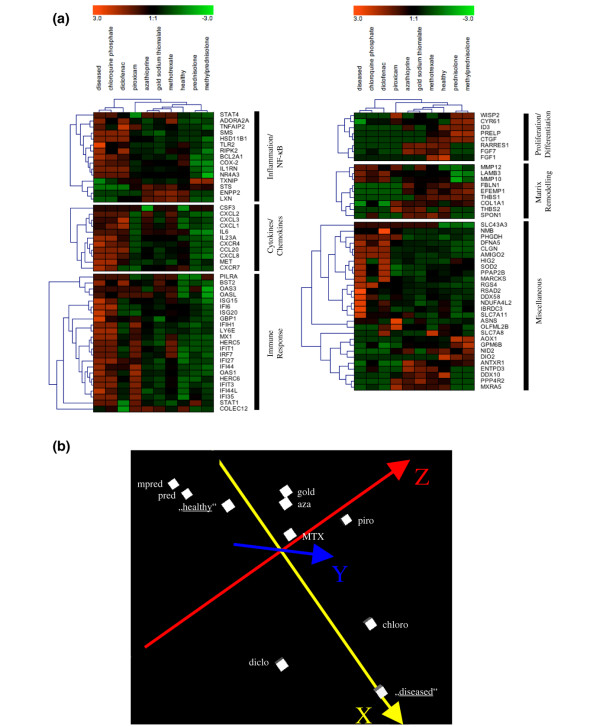
Hierarchical clustering and principal components analyses of rheumatoid arthritis (RA)-related chondrocyte gene expression levels in response to antirheumatic treatment. Hierarchical clustering and principal components analyses of mean expression values of RA-related chondrocyte genes were performed for the 'diseased' status (RASFsn-stimulated), the 'healthy' status (NDSFsn-stimulated), and the drug-treated 'diseased' status (RASFsn antirheumatic drug-stimulated). **(a) **Hierarchical clustering analysis (tree plot). Colours represent relative levels of gene expression: bright red indicates the highest level of expression, and bright green indicates the lowest level of expression. Hierarchical clustering analysis showed that treatment with disease-modifying antirheumatic drugs (DMARDs) methotrexate, azathioprine, and gold sodium thiomalate resulted in chondrocyte expression patterns that were closely related to the 'healthy' status. Chloroquine phosphate and diclofenac treatment had only minor effects because they clustered together with RASFsn-stimulated chondrocytes ('diseased' status). Steroidal anti-inflammatory drug (SAID) treatment reverted the expression of some RA-related genes even beyond the 'healthy' level. **(b) **Principal components analysis (three-dimensional plot) demonstrates the quantitative differences of drug response. DMARDs, except for chloroquine phosphate, and SAIDs reduced the distance between RASFsn and NDSFsn stimulation to a minor difference, whereas DMARDs located toward the 'diseased' status and SAIDs reverted beyond the location of the 'healthy' status. aza, azathioprine; chloro, chloroquine phosphate; diclo, diclofenac; gold, gold sodium thiomalate; mpred, methylprednisolone; MTX, methotrexate; NDSFsn, supernatant of untreated healthy donor synovial fibroblast; NF-κB, nuclear factor-kappa-B; piro, piroxicam; pred, prednisolone; RASFsn, supernatant of untreated rheumatoid arthritis synovial fibroblast.

The effect of antirheumatic drugs on human chondrocytes was very different, ranging from a strong reversion (SAIDs) to minor effects (chloroquine phosphate). To directly compare and to visualise these effects, hierarchical cluster analysis and principal components analysis were performed (Figure 4). Chloroquine phosphate and diclofenac had only minor effects and clustered close to the 'diseased' status of untreated RASFsn-stimulated chondrocytes. In contrast, the DMARDs azathioprine, gold sodium thiomalate, and MTX were much more effective, reverted most of the RA-induced signature, and revealed similar quantitative effects. SAIDs finally displayed highest potency and reverted expression of many genes to 'healthy' levels or even beyond.

### Pathways to stimulate chondrocyte regeneration

The KEGG database was retrieved for the pathways to which the 110 RA-related genes belong. These pathways comprised cytokine-cytokine receptor interaction, Jak-STAT (signal transducer and activator of transcription) signalling, Toll-like receptor (TLR) signalling, transforming growth factor-beta (TGF-β) signalling, focal adhesion, extracellular matrix (ECM) receptor interaction, ether lipid metabolism, and cell communication. Drug-specific dominance of action is summarised in Additional data file 3. The DMARDs azathioprine and gold sodium thiomalate, the NSAID piroxicam, and the SAIDs prednisolone and methylprednisolone targeted numerous RA-related pathways involved in cytokine/chemokine activity (cytokine-cytokine receptor interaction and Jak-STAT signalling), matrix remodelling (focal adhesion, TGF-β signalling, and ECM receptor interaction), and lipid metabolism (ether lipid metabolism, biosynthesis of steroids, and arachidonic acid metabolism). In contrast, chloroquine phosphate and diclofenac had only minor effects on RA-related pathways.

### Validation of microarray data by real-time reverse transcription-polymerase chain reaction and enzyme-linked immunosorbent assay

**Figure 5:**
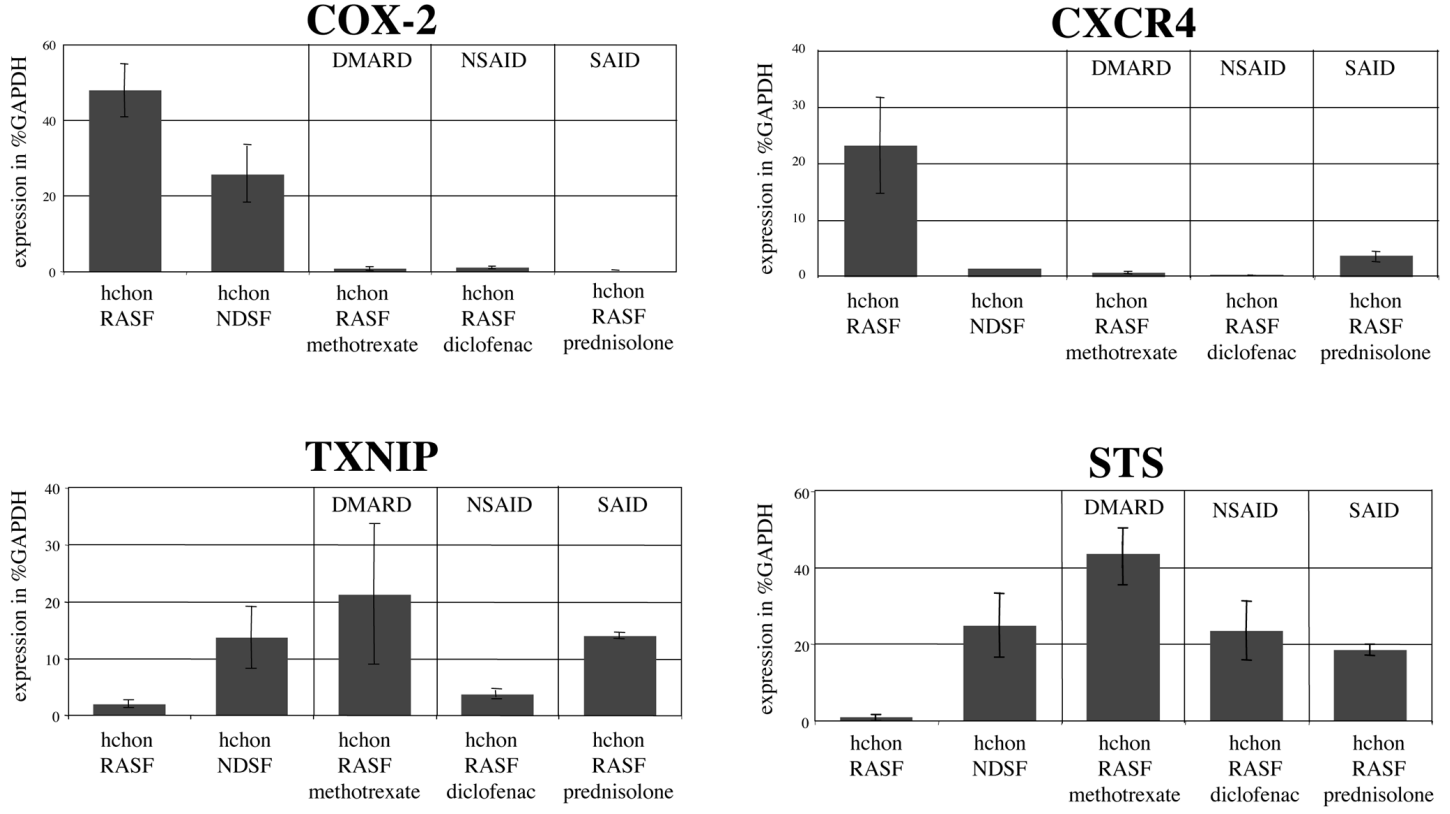
Real-time reverse transcription-polymerase chain reaction (RT-PCR) expression analysis of selected rheumatoid arthritis (RA)-related chondrocyte genes in response to antirheumatic treatment. Real-time RT-PCR confirmed the expression profiles of cyclooxygenase-2 (*COX-2*), chemokine (C-X-C motif) receptor-4 (*CXCR-4*), thioredoxin interacting protein (*TXNIP*), and steroid sulfatase (*STS*) following treatment with methotrexate (disease-modifying antirheumatic drug [DMARD]), diclofenac (nonsteroidal anti-inflammatory drug [NSAID]), and prednisolone (steroidal anti-inflammatory drug [SAID]). Expression of *COX-2 *and *CXCR-4 *was induced in RASFsn-stimulated chondrocytes and repressed again following antirheumatic treatment. Expression of *TXNIP *and *STS *was repressed in RASFsn-stimulated chondrocytes and induced again following antirheumatic treatment. Expression of selected genes was calculated as the percentage of glyceraldehyde 3-phosphate dehydrogenase (*GAPDH*) expression. The mean of each triplicate well is plotted, and the error bars represent the standard deviation. RASFsn, supernatant of untreated rheumatoid arthritis synovial fibroblast.

**Figure 6:**
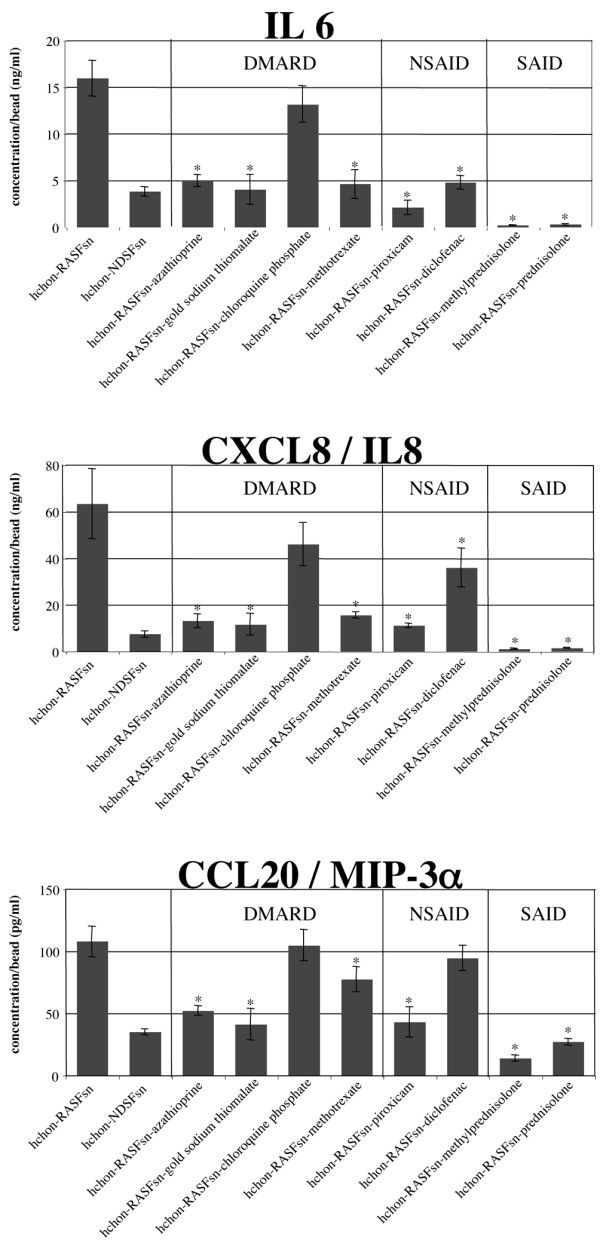
Enzyme-linked immunosorbent assay (ELISA) analysis of selected rheumatoid arthritis (RA)-related chondrocyte protein secretions in response to antirheumatic treatment. ELISA analysis confirmed the expression profiles of interleukin-6 (*IL-6*), interleukin-8 (*CXCL-8*/*IL-8*), and macrophage inflammatory protein-3α (*CCL-20*/*MIP-3α*) following treatment with azathioprine, gold sodium thiomalate, chloroquine phosphate, methotrexate, piroxicam, diclofenac, methylprednisolone, and prednisolone on the protein level. The secretion of the cytokines IL-6, CXCL-8/IL-8, and CCL-20/MIP-3α was induced in RASFsn-stimulated chondrocytes. All examined antirheumatic drugs significantly repressed the synthesis of IL-6 and CXCL-8/IL-8 (except for chloroquine phosphate) and repressed the synthesis of CCL-20/MIP-3α (except for chloroquine phosphate and diclofenac) in human chondrocytes, as already determined by microarray analysis. The mean of each triplicate well is plotted, and the error bars represent the standard deviation. Statistical analysis was performed for chondrocytes stimulated with supernatant of antirheumatically treated rheumatoid arthritis synovial fibroblasts (RASFs) compared the untreated condition (*P < 0.05). DMARD, disease-modifying antirheumatic drug; NSAID, nonsteroidal anti-inflammatory drug; RASFsn, supernatant of untreated rheumatoid arthritis synovial fibroblast; SAID, steroidal anti-inflammatory drug.

To confirm the expression profiles that were determined by microarray analysis, expression of selected genes was verified by real-time RT-PCR (Figure 5) and ELISA (Figure 6). For validation by PCR, two genes with increased expression and two genes with decreased expression after stimulation with RASF supernatant were selected. *COX-2 *as a product involved in proinflammatory actions of the chondrocyte itself was selected because of its broad and differential responsiveness to all drugs, its potent downregulation by glucocorticoids, and its exceptional role in current treatment strategies of rheumatic diseases. *CXCR-4 *as the second upregulated gene with differential response to all drugs is known to sensitise chondrocytes for MMP secretion upon stromal cell-derived factor-1 (SDF-1) stimulation and to be involved in chondrocyte death induction by pathological concentrations of SDF-1 [27,28]. Both genes are well established in chondrocyte pathology and validate the relevance of the *in vitro *model. The two genes *TXNIP *and *STS *are both downregulated after stimulation with RASF supernatant and are not yet described in RA-related cartilage destruction. *TXNIP *is involved in oxidative stress metabolism by inhibiting thioredoxin and thus represents a marker for the potency to response to oxidative stress. *STS *is involved in the biosynthesis of steroids and may be involved in processes of growth and cartilage maturation [29].

PCR validation experiments were performed for representative antirheumatic drugs from the group of DMARDs (MTX), NSAIDs (diclofenac), and SAIDs (prednisolone). Upregulation of *COX-2 *and *CXCR-4 *in chondrocytes by RASFsn stimulation and downregulation upon treatment with MTX, diclofenac, and prednisolone were confirmed. Similarly, regulation of *TXNIP *and *STS *as identified by microarray analysis with a decrease after RASFsn stimulation and an increase after treatment with MTX, diclofenac, and prednisolone was also confirmed by PCR.

ELISA analysis of the supernatants was performed to validate the expression profiles of *IL-6*, the *chemokine (C-X-C motif) ligand-8 *(*CXCL-8/IL-8*), and the *chemokine (C-C motif) ligand-20 *(*CCL-20/MIP-3α*) on the protein level (Figure 6). Cytokines/chemokines are potent mediators of inflammation, and increased chondrocyte expression upon proinflammatory stimulus has been reported. However, a drug-induced suppression of cytokine/chemokine secretion from human chondrocytes has not yet been described and thus was selected for validation.

The protein secretions of IL-6, CXCL-8/IL-8, and CCL-20/MIP-3α were increased in RASFsn-stimulated chondrocytes compared with NDSFsn stimulation. Consistent with the microarray data, treatment with azathioprine, gold sodium thiomalate, MTX, piroxicam, diclofenac, methylprednisolone, and prednisolone resulted in significantly decreased levels of IL-6 and CXCL-8/IL-8. Treatment with chloroquine phosphate did not significantly repress IL-6 and CXCL-8/IL-8 secretion from human chondrocytes. As already determined by microarray analysis, treatment with the examined antirheumatic drugs exclusive of chloroquine phosphate and diclofenac significantly repressed the synthesis of CCL-20/MIP-3α in human chondrocytes. Thus, the gene expression patterns of *IL-6*, *CXCL-8/IL-8*, and *CCL-20/MIP-3α *could be confirmed on the protein level for all antirheumatic drugs examined.

## Discussion

To our knowledge, this is the first genomic study that analysed the molecular mechanisms of RA-related chondrocyte dysfunction and regeneration in response to treatment with commonly used antirheumatic drugs. The study was based on a previously published *in vitro *model [18] that induced gene expression of inflammatory and destructive mediators and repressed regeneration and matrix formation when chondrocyte bead cultures were exposed to the secreted factors of a well-characterised RASF cell line. RASF-stimulated human chondrocytes showed a disturbed homeostasis on the molecular level, and the RA-induced gene expression signatures were considered to be relevant for chondrocyte dysfunction in RA.

In the present study, we show that the different classes of drugs exhibited distinct effects on the RA-induced signature in human chondrocytes. SAIDs were most effective to revert the molecular changes from the 'diseased' to the 'healthy' pattern. SAIDs were followed by the DMARDs azathioprine, gold sodium thiomalate, and MTX, whereas the NSAIDs and the DMARD chloroquine phosphate had the least effects. Thus, the molecular data reflect the clinical experience of therapeutic efficiency. Furthermore, best responses were associated with a strong suppression of proinflammatory cytokines (*IL-6 *and *IL-23A*), chemokines (*CCL-20 *and *CXCL-8*), and *COX-2*. Patterns typical for putative regenerative processes with downregulation by RASF supernatant and reversion or even upregulation upon treatment were seen mostly for matrix genes but also for some factors, which may be inductors of regeneration like *CTGF *[30] or *CYR-61 *[31]. The model that we applied for this study was selected as a compromise of the advantages for standardisation, availability, and comparability with parallel and future testing of different drugs and the disadvantage of an *in vitro *cartilage model and a transformed RASF cell line.

The RASF cell line certainly does not reflect all aspects of the *in vivo *situation and may differ in some aspects from primary fibroblast cultures of RA patients. Nevertheless, previous studies have demonstrated that the RASF cell line is a prototype of activated SFs expressing genes and secreting inflammatory cytokines (for example, IL-1α, IL-1β, IL-6, IL-11, IL-16, IL-18, CXCL-1–3/Gro-α-γ, CXCL-8/IL-8, CCL-2/monocyte chemoattractant protein-1, basic fibroblast growth factor, and leukaemia inhibiting factor) and matrix-degrading enzymes (for example, MMP-1, cathepsin-B, and cathepsin-L) associated with the pathomechanism of RA [14,18,21,22]. The current setting of the model is focused on the effects of RASFs on chondrocyte gene expression and excludes the impact of lymphocytes and macrophages. This has the advantage that effects can be precisely attributed to the secretome of RA fibroblasts. A composed model with several different cell types would impede functional interpretation of the signature, especially with respect to the fact that lymphocyte presence and activity can vary widely from patient to patient and such differences cannot be addressed without knowing the individual components. Thus, stepwise development of such signatures with secretomes of other cell types will account for these additional effects and will help to interpret, at the end, the effects of drugs on more complex settings like cocultures of different cell types or even inflamed tissues. In addition, during recent years, activated RASFs have been determined to be the key players of cartilage destruction in RA by perpetuating the proinflammatory environment in synovial joints and by destroying cartilage matrix and chondrocyte homeostasis [6,7,15]. Therefore, the model reflects major mechanisms related to RA-induced cartilage destruction. The alginate bead culture of chondrocytes was chosen because (a) human chondrocytes could be cultured batchwise in a phenotype-stabilising surrounding, (b) human chondrocytes could be stimulated batchwise with conditioned supernatant of untreated and drug-treated SFs, and (c) total RNA could be isolated easily from human chondrocytes after isolation from the alginate.

The different soluble factors secreted by RASFs were considered to mediate the RA-induced gene expression pattern in human chondrocytes. Treatment of the RASF cell line with antirheumatic drugs was shown to repress many of these proinflammatory factors, with SAIDs being most effective [14]. Therefore, we hypothesise that antirheumatic drugs exert their effects predominantly on RASFs and their secretome but may also act on human chondrocytes directly like SAIDs.

This study disclosed SAIDs to be most effective in reverting RA-induced gene expression in human chondrocytes even beyond the 'healthy' level, in particular the expression of genes associated with inflammation/NF-κB (*BCL2-A1 *and *COX-2*) and cytokine/chemokine activity (*CXCL-8/IL-8 *and *IL-6*). This is consistent with the pathway analysis that revealed SAIDs to target particularly pathways involved in cytokine/chemokine activity and ECM remodelling. SAID treatment of RASFs has been shown to repress the synthesis of the proinflammatory IL-1β and CXCL-8/IL-8 in SFs [14] and thus prevent human chondrocytes from stimulation by these factors. This is in line with the clinical application of SAIDs for intra-articular injection to suppress inflammation and disease activity in the short term. Consistent with our results, general effects of SAIDs include inhibition of the synthesis of inflammatory cytokines, reduction of *COX-2 *expression, and immune suppression [32-34]. Furthermore, glucocorticoid treatment of RA patients has been shown to reduce progression of cartilage destruction [35,36]. Thus, SAID therapy is appropriate to achieve rapid suppression of cartilage destruction and to control symptoms. For long-term treatment, however, adverse effects provoke serious problems [32,33].

Hierarchical clustering and principal components analyses revealed that the DMARDs azathioprine, gold sodium thiomalate, and MTX effectively revert the RA-related chondrocyte gene expression toward the level of 'healthy' expression. Again, genes involved in inflammation/NF-κB signalling, cytokine/chemokine activity, immune response, proliferation/differentiation, and matrix remodelling were predominantly involved. In accordance with these results, DMARDs are described to interfere with the disease process, thereby impeding both the inflammatory and the destructive processes in RA [2]. Among the DMARDs, MTX is regarded as the most effective cornerstone of RA therapy [37,38]. Interestingly, only marginal differences between these three DMARDs were found although their molecular modes of action differ. Azathioprine is a prodrug that is converted via 6-mercaptopurine by a series of transferases, kinases, and reductases to exert cytotoxicity by DNA incorporation and by inhibiting purine *de novo *synthesis [39]. Apparently, RASFs were capable of metabolising azathioprine into active compounds because cytotoxic effects of azathioprine on RASFs were determined by MTS cell proliferation assay (data not shown). MTX is a folate analogue and inhibits methylation processes, and gold sodium thiomalate has a complex pharmacology, which recently was reported to interfere with *COX-2 *transcription [40,41]. *In vivo*, these actions may differ with respect to the cell type (suppression of lymphocyte proliferation, induction of apoptosis in activated T cells and monocytes, and inhibition of macrophage activation) or the kinetics of drug action.

Treatment with chloroquine phosphate, in contrast, resulted in only minor effects. Chloroquine phosphate is an antimalarial drug used in the treatment of RA. The exact mechanism of drug action remains unknown. This study showed that chloroquine phosphate repressed the expression of selected RA-related genes associated with inflammation/NF-κB signalling and immune response. However, the expression of the majority of RA-related genes was not reproducibly influenced following treatment with chloroquine phosphate. In agreement with the results of this study, chloroquine phosphate has been described to fail in inhibiting radiographic joint destruction and to have a relatively slow onset of action when compared with other DMARDs [34,42].

NSAIDs were described to relieve joint swelling but to have only minor effects on disease progression and cartilage breakdown. As symptomatic agents, NSAIDs were supposed to inhibit prostaglandin synthesis by COXs and to help to control symptoms but to fail to retard or even to heal RA-related joint destruction [2,3,43]. This is in line with the results from microarray analysis that determined only minor effects for diclofenac and moderate effects for piroxicam on chondrocyte RA-related gene expression. Piroxicam effects contrasted with those of diclofenac by substantially repressing *COX-2 *expression in chondrocytes. Interestingly, diclofenac treatment of RASFs has been shown to have minor effects on the expression profile of disease-related genes in SFs [14]. Probably, diclofenac exerts the RA-related effects on nonfibroblastic cell types that infiltrate the synovium rather than on SFs [44].

Overall, molecular differences of the various drugs are reflected by the clinical experience that steroids and the three DMARDs MTX, azathioprine, and gold sodium thiomalate, but not chloroquine phosphate or NSAIDs, may be effective in inhibiting radiographic joint destruction [2,3,34,42,43]. Furthermore, SAIDs are best at suppressing the RA-induced changes in chondrocytes but are associated with many side effects. Thus, new therapeutic strategies may focus especially on molecular effects induced by steroids and the effective DMARDs but not induced or insufficiently induced by chloroquine phosphate and NSAIDs. This qualitative difference of drugs is best reflected by the expression pattern of *COX-2 *and several cytokines/chemokines (*IL-6*, *CXCL-8*/*IL-8*, *IL-23A*, and *CCL-20*). Inversely, *CTGF*, *CYR-61*, and *TXNIP *are suppressed by RASFs and reversely induced by the effective drugs.

Inhibition of the transcription of *COX-2* can be considered as more effective than blocking of the enzyme activity. Transcription of *COX-2 *was highest suppressed compared with all other genes by SAIDs, which were also the most effective drugs. Interestingly, prostaglandin E_2 _synergistically with IL-23 was reported to favour human Th17 expansion [45] and prostaglandin E_2 _may also induce IL-23 in bone marrow-derived dendritic cells [46]. Here, we find both increased *COX-2 *and *IL-23 *expression in chondrocytes after stimulation by RASFs, suggesting that chondrocytes may contribute to the development of Th17 cells. This T-helper cell type is discussed in several aspects of proinflammatory activities and may trigger a positive feedback loop via IL-6 [47], another candidate found to be induced in chondrocytes by RASFs and also involved in the differentiation of Th17 cells. This suggests that cartilage, when stimulated by RASF supernatant, may contribute to the proinflammatory network of secondary immune reactions. With *COX-2 *(prostaglandins), *IL-23A*, and *IL-6 *as inductors of this process, targeting these molecules could be favourable.

Concerning induction of regenerative processes, *CTGF *and *CYR-61 *may be potential candidates. CYR-61 is reported as a regulator of chondrogenesis [31] and belongs to the same CCN family of molecules as CTGF [48]. However, CTGF is reported to be involved in many fibrotic processes, including lung [49] and kidney [50], indicating that only local application could be advisable.

As long as a causative therapy of RA is not available, the aim will be to find the appropriate combination of targets that is most effective in remitting or even healing of RA-related cartilage destruction. Since sensitive diagnostic tools to assess cartilage destruction/repair are rare, *in vitro *models for the evaluation of drug efficiency and the identification of potent targets are necessary. Candidate genes that evolved in this study for new therapeutic approaches include suppression of specific immune responses (*COX-2*, *IL-23A*, and *IL-6*) and activation of cartilage regeneration (*CTGF *and *CYR-61*). Further studies are needed to investigate the influence of immune cells on chondrocytes to complete the molecular effects induced by inflammatory processes in arthritis.

## Conclusion

This *in vitro *study provides comprehensive insight into the molecular mechanisms involved in RA-induced chondrocyte dysfunction and in drug-related chondrocyte regeneration. Our findings indicate that RA-relevant stimuli result in the molecular activation of inflammatory and catabolic processes in human chondrocytes that is again reverted by antirheumatic treatment. Molecular differences of the various drugs are reflected by the clinical experience. Furthermore, this study provides evidence of numerous pharmacological marker genes to define and induce chondrocyte integrity and regeneration, including established genes (*COX-2*, *CXCR-4*, *IL-1RN*, *IL-6/8*, *MMP-10/12*, and *TLR-2*) and new genes (*ADORA2A*, *BCL2-A1*, *CTGF*, *CXCR-7*, *CYR-61*, *HSD11B-1*, *IL-23A*, *MARCKS*, *MXRA-5*, *NDUFA4L2*, *NR4A3*, *SMS*, *STS*, *TNFAIP-2*, and *TXNIP*). Candidate genes that evolved in this study for new therapeutic approaches include suppression of specific immune responses (*COX-2*, *IL-23A*, and *IL-6*) and activation of cartilage regeneration (*CTGF *and *CYR-61*). Thus, from the molecular point of view, the present study helps to elucidate the role of chondrocytes during cartilage destruction in RA and during antirheumatic therapy and may contribute to the development of novel therapeutic chondro-protective agents and strategies.

## Abbreviations

ADORA2A: adenosine A2A receptor; BCL2-A1: BCL2-related protein-A1; CCL-20: chemokine (C-C motif) ligand-20; COX: cyclooxygenase; CTGF: connective tissue growth factor; CXCR-4: chemokine (C-X-C motif) receptor-4; CYR-61: cysteine-rich angiogenic inducer-61; DMARD: disease-modifying antirheumatic drug; ECM: extracellular matrix; ELISA: enzyme-linked immunosorbent assay; GAPDH: glyceraldehyde 3-phosphate dehydrogenase; GCOS: GeneChip Operating Software; GEO: Gene Expression Omnibus; HSD11B-1: hydroxysteroid (11-beta) dehydrogenase-1; IC_20_: 20% inhibitory concentration; IL: interleukin; IL-1RN: interleukin-1 receptor antagonist; KEGG: Kyoto Encyclopaedia of Genes and Genomes; MARCKS: myristoylated alanine-rich protein kinase C substrate; MIP-3α: macrophage inflammatory protein-3-alpha; MMP: matrix metalloproteinase; MTS: 3-(4,5-dimethylthiazol-2-yl)-5-(3-carboxymethoxyphenyl)-2-(4-sulfophenyl)-2H-tetrazolium; MTX: methotrexate; MXRA-5: matrix-remodelling associated-5; NDSF: normal (healthy) donor synovial fibroblast; NDSFsn: supernatant of untreated normal (healthy) donor synovial fibroblast; NDUFA4L2: NADH dehydrogenase (ubiquinone) 1 alpha subcomplex, 4-like 2; NF-κB: nuclear factor-kappa-B; NR4A3: nuclear receptor subfamily 4, group A, member 2; NSAID: nonsteroidal anti-inflammatory drug; PCR: polymerase chain reaction; PLA2G2A: phospholipase A2 group IIA; PTX3: pentraxin-related gene; RA: rheumatoid arthritis; RASF: rheumatoid arthritis synovial fibroblast; RASFsn: supernatant of untreated rheumatoid arthritis synovial fibroblast; RIPK2: receptor-interacting serine-threonine kinase 2; RMA: Robust Multichip Analysis; RSAD2: radical S-adenosyl methionine domain containing 2; RT-PCR: reverse transcription-polymerase chain reaction; SAID: steroidal anti-inflammatory drug; SDF-1: stromal cell-derived factor-1; SF: synovial fibroblast; STAT: signal transducer and activator of transcription; STS: steroid sulfatase; TGF-β: transforming growth factor-beta; TLR: Toll-like receptor; TNF: tumour necrosis factor; TNFAIP-2: tumour necrosis factor-alpha-induced protein-2; TXNIP: thioredoxin interacting protein; VCAN: chondroitin sulfate proteoglycan 2; WISP2: WNT1 inducible signalling protein 2.

## Competing interests

CK is an employee of TransTissueTechnologies GmbH (Berlin, Germany). CK, TH, and MS hold a patent related to the content of this manuscript: 'An *in vitro *cell interaction culture system for testing and developing medicaments' (DE 10 10 54 20 B4). MS is a shareholder of CellServe GmbH (Berlin, Germany) and works as a consultant for BioTissue Technologies AG (Freiburg, Germany). The other authors declare that they have no competing interests.

## Authors' contributions

KA helped to perform the gene expression data processing, participated in the design and coordination of the study, helped to draft the manuscript, and helped to conduct the cell culture experiments and to perform the microarray and ELISA experiments. TH helped to perform the gene expression data processing, participated in the design and coordination of the study, and helped to draft the manuscript. KA and TH contributed equally to this article. CL helped to perform the gene expression data processing, participated in the design and coordination of the study, and shared responsibility for collection, assembly, and analysis of data and data interpretation. JR helped to perform the gene expression data processing and participated in the design and coordination of the study. LM shared responsibility for collection, assembly, and analysis of data and data interpretation. AW helped to conduct the cell culture experiments and to perform the microarray and ELISA experiments. MS and CK helped to conceive the study and participated in its design and coordination. All authors read and approved the final manuscript.
